# Direction of Movement Is Encoded in the Human Primary Motor Cortex

**DOI:** 10.1371/journal.pone.0027838

**Published:** 2011-11-16

**Authors:** Carolien M. Toxopeus, Bauke M. de Jong, Gopal Valsan, Bernard A. Conway, Klaus L. Leenders, Natasha M. Maurits

**Affiliations:** 1 Department of Neurology, University Medical Center Groningen, University of Groningen, Groningen, The Netherlands; 2 Bioengineering Unit, University of Strathclyde, Glasgow, United Kingdom; The University of Western Ontario, Canada

## Abstract

The present study investigated how direction of hand movement, which is a well-described parameter in cerebral organization of motor control, is incorporated in the somatotopic representation of the manual effector system in the human primary motor cortex (M1). Using functional magnetic resonance imaging (fMRI) and a manual step-tracking task we found that activation patterns related to movement in different directions were spatially disjoint within the representation area of the hand on M1. Foci of activation related to specific movement directions were segregated within the M1 hand area; activation related to direction 0° (right) was located most laterally/superficially, whereas directions 180° (left) and 270° (down) elicited activation more medially within the hand area. Activation related to direction 90° was located between the other directions. Moreover, by investigating differences between activations related to movement along the horizontal (0°+180°) and vertical (90°+270°) axis, we found that activation related to the horizontal axis was located more anterolaterally/dorsally in M1 than for the vertical axis, supporting that activations related to individual movement directions are direction- and not muscle related. Our results of spatially segregated direction-related activations in M1 are in accordance with findings of recent fMRI studies on neural encoding of direction in human M1. Our results thus provide further evidence for a direct link between direction as an organizational principle in sensorimotor transformation and movement execution coded by effector representations in M1.

## Introduction

Dynamic interaction with the outside world requires the translation of several modalities of sensory information into the appropriate motor output [1]. Although it is not clear how the brain achieves this translation, the final motor commands are thought to be partly based on extrinsic parameters derived from a visuospatial framework [1]–[4]. A first step in the interaction with an object is to locate where the object is relative to the position of the body and surrounding objects by mapping the peripersonal space [5]. This mapping is thought to be obtained by a transformation of the visually acquired image of the environment into three axes; a horizontal, a vertical and an in front-behind-us axis (or ‘depth’) [6]. In parallel, facilitated by parieto-occipital networks, proprioceptive information is integrated in the visually acquired image, resulting in a three-dimensional map serving as an egocentric visuospatial reference frame providing directional vectors for goal-directed movement [1], [7]–[14]. Based on this view, direction of movement is an important factor in translating complex multisensory information into parameters used for motor planning- and execution [15], [16]. Although directional parameters were demonstrated to be derived from visuospatial information, which is facilitated primarily by frontoparietal networks [12], [17]–[19], how direction of movement is further used in the motor cortex for planning and execution of final motor commands remains to be elucidated.

The question of how the brain uses functional parameters in movement execution was already addressed by J.H. Jackson in the late nineteenth century [20]. At present, although a somatotopic organization of the human primary motor cortex [M1] is well-established [21], a solely somatotopic organization of M1 has been recognized as a simplified view on functional organization of the brain considering the substantial overlap in representation of adjacent body segments [22]–[24]. Hence, other parameters may play a role in generating appropriate motor output. Yet, it is unclear how functional parameters such as direction of movement are represented in M1 and how they are used for computation of final motor commands [1], [4], [25], [26].

In the past decades, direction was identified as a specific functional parameter, particularly in studies using single cell recordings in non-human primates [15], [27], [28]. These studies concluded that populations of neurons in the premotor cortex (PMC) and M1 are directionally tuned [2], [28]. Furthermore, it has been proposed that populations of neurons with a preferred directional tuning are spatially mapped along the cortical surface [29], suggesting the presence of a neural substrate relating direction to the (final) execution of goal-directed movement in M1 [8], [15], [28], [30], [31]. Besides single cell recordings in non-human primates, only recently human studies with functional magnetic resonance imaging (fMRI) concluded that in M1 clusters of neurons are tuned directionally [32], [33]. It was consequently suggested that, in order to translate complex sensorimotor information into muscle activity patterns, also in humans direction may be used as a unifying sensorimotor parameter encoded within M1 [32], [33]. In addition to these human studies, the present study aimed to explore how direction of hand movement is incorporated in the representation of the manual effector system in M1 by employing fMRI and a centre-out step-tracking task [34]. We hypothesized that by employing fMRI and a simple manual step-tracking task, we will be able to reproduce findings of the recently published fMRI studies and determine a neural substrate encoding for direction within M1. Furthermore, we expect direction-related activations to be mapped in a spatially segregated fashion within the hand/arm representation in M1. Such direction-related activation patterns will provide evidence for a link between direction as an organizational principle in visuomotor transformation and subsequent movement execution coded by the specific effector representations in M1.

## Materials and Methods

### Ethics statement

The study was approved by the Medical Ethical Committee of the University Medical Center Groningen. Subjects participated after full explanation of the study and having given written informed consent in accordance with the Declaration of Helsinki (2008) prior to participation.

### Subjects

Subjects had to be right handed as assessed by the Annet Handedness Scale [35]. Exclusion criteria were a history of epileptic seizures, head injury, neurological diseases, psychiatric diseases or the use of any type of medication affecting the central nervous system (CNS). Subjects came for two visits on separate days with a maximum interval of two weeks. During the first visit subjects were screened neurologically. A total of nineteen right-handed healthy subjects participated. One subject was excluded due to an anomaly on the T1 anatomy scan. Eighteen healthy subjects entered the fMRI analysis (age range: 51–69, mean 58.7±5.4 (SD)). The present experiment was part of a larger study on differences in the cerebral organization of movement between patients with Parkinson's disease and healthy subjects. Therefore all subjects in the present study were elderly.

### Task

**Figure 1:**
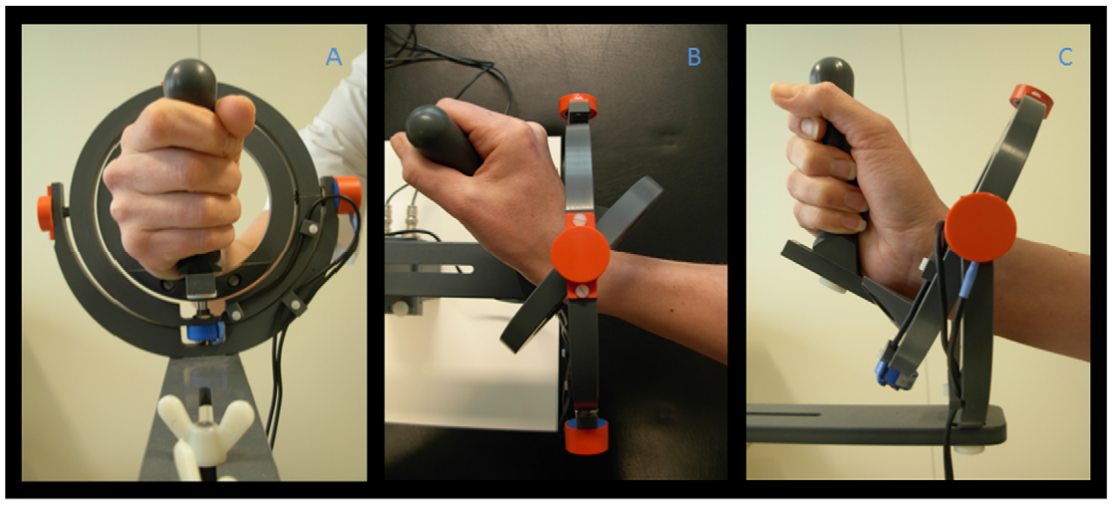
Photograph of the wrist manipulandum. The construction consists of two concentric rings that move around perpendicular axes and allow two degrees of freedom for wrist movement: wrist flexion-extension, ulnar-radial deviation and all combinations thereof. a: (frontal view) neutral position (origin), the right hand is positioned in a vertical plane, holding the grip of the manipulandum; b: (top view) full wrist extension and c: (side view) full radial deviation.

All subjects performed a visual step-tracking task using a magnetic resonance (MR) compatible manipulandum similar to the manipulandum used by Hoffman and Strick for their studies on step-tracking [34] (fig. 1). The manipulandum that was used is a joystick-like device that is able to rotate in two perpendicular planes allowing wrist flexion-extension, wrist ulnar-radial deviation and all combinations thereof. The right wrist joint was positioned in the center of the two concentric rings composing the device, holding the grip of the manipulandum (thumb on top). The fingers were taped to the thumb to remind subjects to hold the grip of the manipulandum with all fingers. The manipulandum was mounted on the right side of the MR table and was carefully adjusted to optimally fit in the scanner. It was ensured that the subjects were able to move freely in each direction. To provide visual feedback on task performance, angular displacement was measured in both planes by two potentiometers (X and Y) integrated in the manipulandum and displayed as a cursor (a 5×5 mm closed square) on a screen. During acquisition of fMRI scans subjects watched the task and visual feedback on their performance on a screen (display dimensions 44×34 cm) which was projected by a beamer (resolution 1024×768 pixels, Barco, Belgium) on a mirror placed at a distance of 11 cm from the face. The distance between screen and mirror was 64 cm. If necessary, MR compatible glasses were available to correct visual acuity. Target stimuli were generated using Spike 2 (*CED, UK*) and an analog-to-digital converter board (*Power 1401, Cambridge Electronic Design (CED), Cambridge, U.K.*).

**Figure 2:**
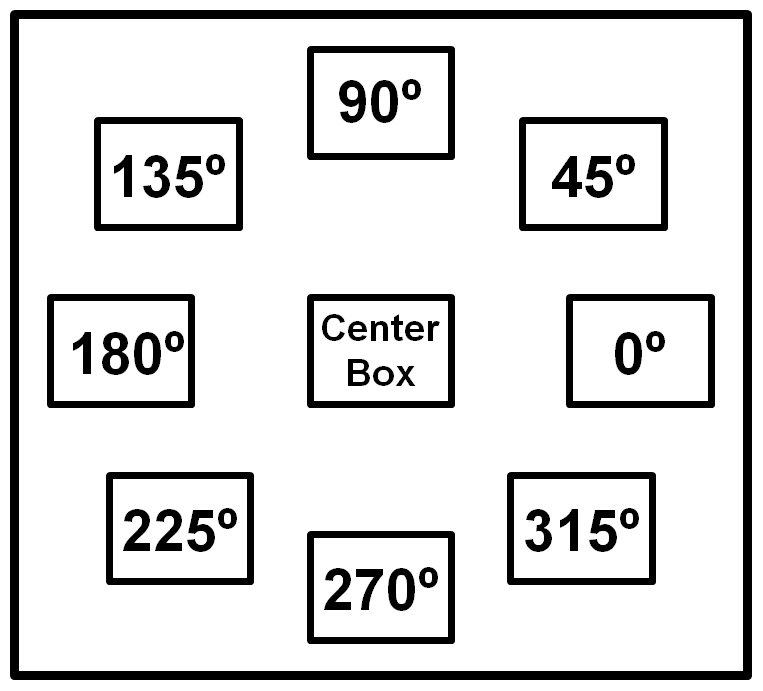
Schematic overview of the eight directional stimuli. Orientation of eight step-tracking directions: 0°, 45°, 90°, 135°, 180°, 225°, 270°, 315° and 360°. The center box is the start and end point of each step-track movement.

Prior to task execution, subjects placed their cursor in the ‘center box’ (3×1.5 cm open square) corresponding to a neutral wrist position. A warning cross preceding appearance of the target was displayed in this center box for 1 second. After disappearance of the warning cross, a target stimulus (3×1.5 cm open square) appeared at one of 8 possible positions (fig. 2). All eight directional stimuli had the same distance (20 degrees) relative to the center of the screen and were equally spaced. Regarding the hand position in the manipulandum, movements in directions 0° and 180° corresponded with extension and flexion, respectively whereas movements in directions 90° and 270° corresponded with radial and ulnar deviation, respectively. These four directions are further referred to as main directions. The remaining directions (45°, 135°, 225° and 315°) required combinations of flexion-extension and radial-ulnar deviation and, therefore, are further referred to as intermediate directions. Subjects were instructed to move the cursor, controlled by the manipulandum, as fast as possible to the target. After moving towards the target, subjects had to hold the cursor in the target box until it disappeared (3 seconds after appearance of the target stimulus) before returning (smoothly) to the center box. Each step-track movement lasted 5 seconds. After every 10 step-tracks, there was a break of 4 seconds. Each step-track block consisted of 40 stimuli, 5 in each of the 8 different directions presented in fixed randomized order (randomized, but the order was the same for each subject). Subjects performed four blocks of the step-tracking task.

During the first visit, subjects practised the task in a sitting position for at least four blocks and in a dummy MR scanner for at least one block. Just before the scan session during the second visit, the task was shortly practised again (for less than one block) to ensure that all subjects remembered task instructions and were able to execute the task during the fMRI session. Online monitoring of task performance was enabled by a computer screen in the MR control room. When required, subjects were given auditory feedback between task blocks.

### MRI characteristics

Data acquisition was performed using a 3 Tesla Magnetic Resonance System (Philips, Best, The Netherlands) with a standard 6 channel head coil. T2* weighted, 3D functional images were obtained using multislice echo planar imaging (EPI) with an echo time (TE) of 30 ms and a repetition time (TR) of 2000 ms. Per TR 39 axial slices, field of view (FOV) 224 mm, with a 64×64 matrix and isotropic voxel size of 3.5×3.5×3.5 mm were acquired. The functional scan included 106 volumes per block. Additional T1-weighted three-dimensional anatomical scans with an axial orientation and a matrix size of 256×256 mm were obtained to provide anatomical information (isotropic voxel size 1×1×1 mm).

### Data analysis

Processing of images and statistical analyses were conducted with Statistical Parametric Mapping (SPM) version 5 (2005, Wellcome Department of Cognitive Neurology, London, UK; http://www.fil.ion.ucl.ac.uk/spm). Pre-processing included standard slice time correction, realignment and co-registration of functional and anatomical scans. Images were normalized to the template of the Montreal Neurological Institute (MNI) and smoothed using a Gaussian filter of 8 mm full width at half maximum (FWHM). Data was detrended using the standard SPM high-pass filtering with a cut-off at of 128 Hz. Analyses were time-locked to the eight different stimuli to enable event-related analysis. Brain activations were computed according to the standard statistical procedures in SPM. Statistical parametric maps per subject (first level analysis) were derived using a linear multiple regression model with 8 event-related regressors (one for each direction) and movement parameters as regressors of no interest to account for head movement-related effects [36]. Activation maps (for each movement direction) were entered in an ANOVA (flexible factorial design) with eight levels (corresponding with the eight directions) to investigate cerebral activation patterns related to direction on a group level.

We used whole brain analysis to identify activation patterns related to direction in M1. In order to prevent filtering out subtle activation differences related to directions that might be dominated by overlap in the representation of the hand in M1, we did not use direct comparisons between the different movement directions. Instead, we assessed maximally activated voxels per individual direction. We used false discovery rate (FDR) correction at a threshold of p = 0.01 to limit the number of false positive voxels. To gain insight in segregated/overlapping activations of directions, results were further (visually) assessed using overlays in MRICron [37] at a threshold of T = 3.4 (equivalent to p = 0.01, FDR corrected).

Since the present study did not vary between hand positions during the task, M1 activations related to the various directions resulted from the activity of distinct muscle couples. Therefore, activations in M1 might be attributed to the representation of these muscles instead of being direction-related [8]. In order to dissociate between direction-related and muscle-related activations in M1, we determined activations related to movements along the horizontal and vertical axes. By pooling the two opposite directions for each axis, contributions by individual muscles are ‘averaged out’. Therefore, a difference between these axes in M1 will provide an argument against our findings being muscle representations. Here, activations related to movement along the vertical axis were obtained by pooling directions 90° and 270° and to obtain activation related to movement along the horizontal axes directions 0° and 180° were pooled. We used exclusive masks (masking threshold: p = 0.001) to compare the two main axes at thresholds of p = 0.01 (FDR corrected). Note that exclusive masks remove all voxels reaching significance in one contrast that overlap with the significant voxels in the other contrast. Results were visually compared in MRICron, at thresholds of T = 3.1 (equivalent to p = 0.01, FDR corrected).

## Results

### Direction-related activation in the primary motor cortex [M1]

**Figure 3:**
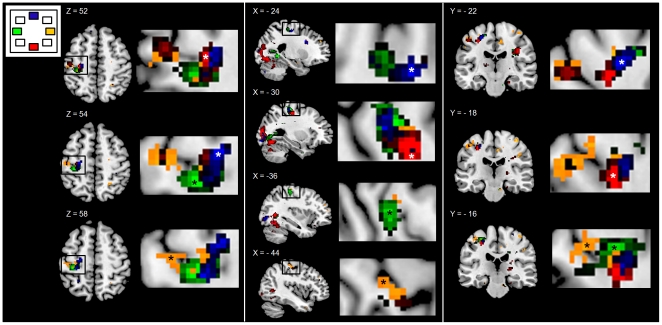
Direction-related activation patterns in the primary motor area. Activation patterns in the primary motor area (M1) along the central sulcus, for movements in the main directions (0°–90°–180°–270°). Activations are shown in three orientations: left panel: axial slices: Z: 52, 54, 56), middle panel: sagittal slices X: −24, −30, −36, −44) and right panel: coronal slices Y: −22, −18, −16). Magnifications of foci of activations are shown at the left side of each slice. In order to identify foci of activations, T-maps of individual directions were plotted at T = 3.4 (equivalent to p = 0.01, corrected) for all four directions. Yellow: 0°, blue: 90°, green: 180° and red: 270°. * =  foci of activation per direction. L  =  left.

**Figure 4:**
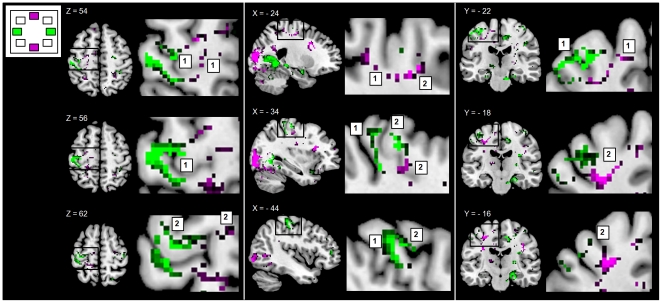
Activations related to movements along the horizontal and vertical axes. Activations related to these axes were compared using exclusive masking (p<0.001). Higher activations for the horizontal axis than vertical axis are indicated in green and higher activations for the vertical axis than horizontal axis are indicated in purple (all activations are plotted with a threshold of T = 3.2 (p<0.01, FDR corrected.). Activations are shown in three orientations: left panel: axial slices: Z: 54, 56, 62), middle panel: sagittal slices X: −24, −34, −44) and right panel: coronal slices Y: −22, −18, −16). Magnifications of foci of activations are shown at the left side of each slice. 1 =  activation in the primary motor cortex (M1), 2 =  activations in the premotor cortex (PMC). L =  left.

For the remainder of this report, activation due to movement of which the direction is specified by the location of a preceding visual cue, is simply referred to as activation related to that direction. Foci of maximum activation were assessed in M1 (Brodmann area (BA) 4). Right hand movement, regardless of its direction, elicited brain activation in the somatotopic representation of the hand in contralateral M1 [21], [22], [24]. However, there were clear differences in the focus of maximum brain activation between the four main directions (0°, 90°, 180° and 270°) (fig. 3–4). Activation related to movements in direction 0° was located most laterally, extending to the cortical convexity, whereas directions 180° and 270° showed activation more medially within the hand area. In addition, we found that the 0° direction also activated ipsilateral M1. The 270° direction elicited activation more ventrally than the other directions whereas activation related to the 90° direction was located between 0° and 180°/270° directions and more dorsally in the M1 hand area. For movements in intermediate directions (225°, 45°, 315°), with the exception of 135°, activations had a less clear focus of activation and were not further analyzed.

### Representations of movement along the vertical and horizontal axes in M1 and PMC

Although the two axes of direction both evoked segregated activations in M1 (fig. 4A), movement along the horizontal axis resulted in more activation (i.e. the activation pattern was more extensive) in M1 than movement along the vertical axis. Moreover, activation related to the horizontal axis extended more towards the anterior surface of M1 (max. activation at −44, −24, 56, p = 0.01 (FDR corrected)), while activation related to the vertical axis was located deeper in the cortex (max. activation at −28, −20, 46, p = 0.030 (FDR corrected)). Thus, activation related to the horizontal axis was located more anterolaterally and dorsally in M1 than activation associated with movement along the vertical axis. Given the direction-sensitivity of the dorsal PMC [28] and its involvement in using visuospatial information for motor preparation [6], [10], [11], we were interested to see whether movements along the horizontal axis might similarly show a larger representation in the PMC. Here, although we found that the extent of activation related to the horizontal axis in the PMC was comparable to that for the vertical axis, we found that PMC activation related to movement along the horizontal axis was localized more latero-posteriorly and closer to M1 (max. activation at −32, −24, 62, p = 0.004 (cluster corrected)) compared to the antero-medial activation related to the vertical axis (max. activation at −24, −12, 54, p = 0.002 (cluster corrected)) (fig. 4B). Additionally, ipsilateral M1/PMC activations related to the horizontal axis were more extensive and more laterally localized compared to the vertical axis.

## Discussion

The present fMRI study indicates that activations related to the performance of movement in specific directions are spatially disjoint within the hand representation in M1. The observed spatial segregation of direction-related activations was most clear for the two directions of movement along each of the principle, i.e. horizontal and vertical, axes of orientation (0°/180° and 90°/270°). The findings of the present study are in accordance with previous studies using single-cell recordings in non-human primates in which neurons and populations of neurons in M1 were found to be directionally tuned [8], [15], [28], [30], [31] in a spatially segregated pattern [29] as well as recent fMRI studies investigating neuronal representation of direction in humans [32], [33]. Therefore, our finding of direction-related activation in the human M1 hand-area extends the concept of M1 functions: aside from a somatotopically arranged effector system, M1 is also involved in higher-order information processing.

The previously described expression of complex stereotypic movements by stimulating the motor cortex in non-human primates already provided arguments for the use of higher order parameters in M1 [38], although the recent study by Griffin et al. concluded to be careful with this interpretation because these movement effects are possibly driven solely by stimulation [39]. Besides the somatotopical arrangement in M1 a functional division can be made in human M1 by demarcating areas 4a (anterior) and 4p (posterior). Binkofski et al. (2002) reported movement-related activation in the superficially located motor area 4a to remain unaffected by attention modulation, while 4p activation – located in the depth of the central sulcus – decreased during visual distraction [40]. Stenekes et al. (2006) observed that 4a was particularly implicated in finger flexion, in contrast to finger extension [41]. They used this finding to re-interpret the results by Binkofski et al., by proposing that increased activation in 4a reflected stronger coupling with sensory information. Such sensorimotor anchoring associated with particularly finger flexion, as well as the focus of activation adjoining the premotor cortex in the study by Stenekes et al., underscored the idea that finger flexion is more dominantly involved in hand functions such as grasping than finger extension. Using a similar explanation, Terumitsu et al. (2009) et al., regarded 4a as a secondary M1 area involved in movement requiring more complex sensory information [42]. The present observation of a larger representation of movements along the horizontal axis in the 4a segment of M1 may, therefore, support the concept that movement along this axis involves increased demand on sensorimotor integration. Indeed, when the two hands work together on a specific task, which involves both hemispheres, actions are predominantly performed in a horizontal plane. Alternatively, although responses were time-locked by the stimuli, one cannot exclude subtle differences in execution of movement related to the horizontal and vertical axes, such as differences in movement speed, that might have contributed to the observed differences in activation patterns between both axes [43]. Nevertheless, given the simplicity of hand movement in our task, in which direction conditions were considered to be balanced for cognitive demands, and the dominant arrangement of a right and left hand working together in task performance, this hypothesis seems plausible [42].

A strong association between direction and the final execution of movements can also be inferred from behavioural studies. For example, Post et al. (2009) found that when hands are positioned asymmetrically, with the volar side of the right hand facing the left thumb, abduction of the right finger is associated with muscle contractions in the contralateral finger that do not induce abduction, but result in movement parallel to the movement direction of the ipsilateral finger [44]. These findings suggest the use of direction-related parameters rather than muscle-related parameters in movement execution. This was also suggested by Z'Graggen et al., (2009) who found that the direction of (thumb) movements evoked by transcranial magnetic stimulation (TMS) were spatially segregated in M1 [45]. These findings are in line with the segregated representation of different directions of movement found in the present study [46].

A few issues have to be taken into account when interpreting our results. First, there are two important differences between the single cell recordings used in non-human primates and fMRI in humans; (i) fMRI measures the blood oxygen level dependent (BOLD) signal, which is not a direct neurophysiological measurement of neuronal activity, although it does provide an index for regional neuronal activity [47]–[49] and (ii) fMRI enables detection of regional changes in cortical activity at the macroscopic level and not at the single neuron, i.e. sub-millimetre, level [32]. This relatively low spatial resolution is a reason to remain cautious regarding final interpretations of fMRI results. Yet, our results are in accordance with primate studies concluding that different directions are encoded by spatially segregated populations of neurons rather than by single neurons [29], [50]. Our results are further consistent with recent fMRI studies that also found movement direction encoded in human M1 [32], [33], [51]. Second, we studied elderly subjects. Although aging may induce changes in movement execution [52], our subjects were younger (mean age: 58.7) than elderly subjects participating in typical studies addressing age-related changes in movement execution. Finally, as the segregation of M1 activations related to the various directions resulted from the activity of distinct muscle couples, one might oppose that this spatial distinction simply reflects the representation of these muscles [53]–[55]. However, the characteristic representation of movement along the horizontal and vertical axes, each obtained by a succession of opposite muscle activities, provides a strong argument against an invariant muscle representation. This finding is consistent with the observations that cortical neurons controlling a single muscle are broadly distributed in M1 [56], [57]. A consequence of broadly distributed cortical neurons, and, thus, the variance in functional connections between the cortical and spinal motor neurons, is that commands from M1 to distinct muscles may be more task-related than muscle-related [8], [58]. Indeed, such neuronal organization within M1 enables the transfer of a higher-order parameter such as direction onto the final commands for specific muscle use.

In order to gain further insight in the use of direction for translating complex multisensory information into muscle activation patterns, neuroimaging methods would benefit from combined assessment with electromyography. From such combined functional brain and quantification of movement information one might obtain evidence to test the hypothesis [15], [38] that direction is used as a functional parameter to select temporal patterns of distinct agonist-antagonist co-activations rather than to separately activate muscles. One might thus speculate that patterns of agonist-antagonist activations are linked to movement in specific directions. Although representation of direction in M1 points at the importance of an *external* parameter, the computation of final motor commands for goal-directed movement in M1 additionally requires integration of *intrinsic* parameters as posture providing information on the potential roles of muscles as either agonists or antagonists [8]. Therefore, it is suggested that M1 is involved in higher order motor control by processing both intrinsic and extrinsic parameters to select the appropriate motor commands.

### Conclusion

In conclusion, the present study demonstrates that distinct activation patterns are related to movement in different directions within the somatotopical representation of the hand in M1 by using fMRI and a step-tracking task. Our findings on direction of movement being encoded in M1 confirm and further specify findings of previous studies on neural encoding of direction of movement in both primates and human. In this way, evidence accumulates for a direct link between direction as an organizational principle in sensorimotor transformation and movement execution coded by effector representations in M1.
